# Association of autism diagnosis and polygenic scores with eating disorder severity

**DOI:** 10.1002/erv.2941

**Published:** 2022-07-19

**Authors:** Ruyue Zhang, Andreas Birgegård, Bengt Fundín, Mikael Landén, Laura M. Thornton, Cynthia M. Bulik, Lisa Dinkler

**Affiliations:** ^1^ Department of Medical Epidemiology and Biostatistics Karolinska Institutet Stockholm Sweden; ^2^ Institute of Neuroscience and Physiology Sahlgrenska Academy Gothenburg University Gothenburg Sweden; ^3^ Department of Psychiatry University of North Carolina at Chapel Hill Chapel Hill North Carolina USA; ^4^ Department of Nutrition University of North Carolina at Chapel Hill Chapel Hill North Carolina USA

**Keywords:** anorexia nervosa, autism spectrum disorder, clinical outcome, comorbidity, polygenic risk score

## Abstract

Among individuals with eating disorders (ED), those with co‐occurring autism are often considered to have more severe presentations and poorer prognosis. However, previous findings have been contradictory and limited by small sample size and/or cross‐sectional assessment of autistic traits. We examine the hypothesis that autism diagnosis and autism polygenic score (PGS) are associated with increased ED severity in a large ED cohort using a broad range of ED severity indicators. Our cohort included 3189 individuals (64 males) born 1977–2000 with current or previous anorexia nervosa who participated in the Anorexia Nervosa Genetics Initiative‐Sweden (ANGI‐SE) and for whom genotypes and linkage to national registers were available. We identified 134 (4.2%) individuals with registered autism diagnoses. Individuals with confirmed autism diagnosis had significantly more severe ED across three sets of severity indicators. Some of the largest effects were found for the proportion of individuals who attempted suicide and who received tube feeding (higher in autism), and for the time spent in inpatient care (longer in autism). Results for autism PGS were not statistically significant. Adapting ED treatment to the needs of individuals with co‐occurring autism is an important research direction to improve treatment outcome in this group.

## Key points

- Among 3189 Swedish individuals with current or previous anorexia nervosa, those with confirmed autism diagnosis (4.2%) experienced higher eating disorder severity across 27 out of 29 indicators.
- Some of the highest risk increases were found for having attempted suicide, having received tube feeding, and time spent in inpatient care for eating disorders.
- Repeating the analyses with autism polygenic score instead of autism diagnosis yielded non‐statistically significant results for all 29 eating disorder severity indicators.

**Abbreviations**
ADHD: attention deficit hyperactivity disorder
AN: anorexia nervosa
AN‐BP: anorexia nervosa binge‐eating/purging type
ANGI‐SE: Anorexia Nervosa Genetics Initiative‐Sweden
BMI: body mass index
CGI: clinical global impression rating scale
CI: confidence interval
CIA: clinical impairment assessment
ED: eating disorder
ED100K: questionnaire used in ANGI‐SE to determine lifetime AN diagnosis
EDE‐Q: eating disorder examination‐questionnaire
GAF: global assessment of functioning
IRR: incidence rate ratio
NDD: neurodevelopmental disorder
NPR: national Patient Register
OED: other eating disorder
OR: odds ratio
PGS: polygenic score
Riksät/Stepwise: Swedish quality registers for specialised eating disorder treatment

## 1 INTRODUCTION

Eating disorders (EDs) and autism co‐occur more frequently than expected by chance (Westwood & Tchanturia, 2017). The association appears to be strongest with anorexia nervosa (AN), but also other EDs (OED) such as bulimia nervosa and binge‐eating disorder are overrepresented in individuals with autism, as are weight extremes (underweight *and* obesity) (Nickel et al., 2019; Sedgewick et al., 2020; Westwood & Tchanturia, 2017). Among individuals with ED, those with co‐occurring autism are often considered to have more severe and enduring ED presentations (i.e., poorer prognosis), and efforts have been made to adapt ED treatment specifically to the needs of individuals with co‐occurring autism (Li et al., 2021; Tchanturia et al., 2020). However, the evidence supporting the association of autism with increased ED severity is marked by weak findings and contradictory results, which we will summarise below.

The majority of studies that explored **ED symptoms at admission for ED treatment** (most often measured with the Eating Disorder Examination‐Questionnaire [EDE‐Q]; Fairburn & Beglin, 2008) did not observe significant associations with autistic traits (Huke et al., 2014; Nazar et al., 2018; Stewart et al., 2017; Tchanturia et al., 2013; Westwood et al., 2018). Only one study reported a significant association of autistic traits with higher EDE‐Q global score (Tchanturia et al., 2019), and another study reported elevations on the subscales *weight concern* and *restraint* with *p*‐values <0.10 (Westwood et al., 2017). None of the identified studies reported a significant difference in body mass index (BMI) at admission (Huke et al., 2014; Tchanturia et al., 2019; Westwood et al., 2017, 2018) or in lowest BMI (Nazar et al., 2018) between individuals with and individuals without autistic traits. No significant differences were found in ED duration (Westwood et al., 2017, 2018) or age of onset (Nazar et al., 2018); however, in the Westwood et al. (2018) sample, individuals with AN who also had autism were on average 1 year younger, corresponding to a Cohen's *d* of 0.6 (*p* = 0.077).

Findings regarding the association between autism and **improvement after ED treatment** are inconsistent. Stewart et al. (2017) reported less improvement on the EDE‐Q *weight concern* and *shape concern* scales in those with high autistic traits, whereas autistic traits did not affect improvement of ED symptoms in Nazar et al. (2018); nor were autistic traits associated with improvement in BMI (Nazar et al., 2018; Tchanturia et al., 2019). Contradictory results also exist in explorations of the impact of high autistic traits on improvement of cognitive flexibility/set‐shifting and central coherence/attention to detail after Cognitive Remediation Therapy (Dandil et al., 2020; Giombini et al., 2022; Tchanturia et al., 2016).

In contrast to these areas, studies examining **ED treatment utilisation** have consistently shown increased risk of receiving inpatient treatment (Nazar et al., 2018; Stewart et al., 2017), involuntary treatment (Clausen et al., 2018), and longer duration of hospital treatment (Tchanturia et al., 2021) for patients with high autistic traits or diagnosed autism. Autistic traits did not seem to affect treatment adherence in one study (Nazar et al., 2018), although another study reported a tendency towards autism potentially *increasing* treatment adherence (non‐statistically significant) (Huke et al., 2014).

Only one group has investigated the effect of autistics traits and autism on the **long‐term outcome** of ED. Four follow‐ups were conducted on the same sample with teenage onset AN (after 6, 10, 18 and 30 years). Autism was consistently associated with poorer outcome in terms of overall mental health, social functioning, employment, socioeconomic status, and Global Assessment of Functioning (Dobrescu et al., 2020; Nielsen et al., 2015, 2022). With regard to ED core symptoms, improvement in dietary restriction over time was limited to the group without autism at both the 18‐year and 30‐year follow‐ups (Nielsen et al., 2015, 2022), possibly reflecting the fact that restrictive/selective eating is common in individuals with autism from childhood and throughout life, and often not related to body image concerns (Kuschner et al., 2015). At the 18‐year follow‐up, improvements in body weight and menstrual functioning were also limited to the group without autism (Nielsen et al., 2015), whereas this effect was not observed at the 30‐year follow‐up (Nielsen et al., 2022).

**In summary,** previous studies among people with eating disorders reported few consistently observed differences between those with high and those with low autistic traits. However, many of the studies were limited by small sample size impacting power to detect effects, as indicated by large effect sizes with non‐statistically significant *p*‐values (e.g., Westwood et al., 2017; Westwood et al., 2018). Another important limitation of previous research is the largely cross‐sectional assessment of autism in most studies, as it is very difficult to separate ‘true’ autistic traits from ‘AN traits’ and from effects of starvation during the acute ill‐state of AN (consider e.g., extreme rigidity, obsessive interests, and social withdrawal). When studying associations between autism and ED, it is therefore preferable to combine cross‐sectional assessments with measures of developmental history, for instance, via parent‐reports of childhood autistic traits, as is done when diagnosing autism in clinical contexts. However, previous studies primarily relied on cross‐sectional, parent‐ or self‐reported measures of autistic traits such as the Autism Spectrum Quotient (AQ‐50 & AQ‐10; Baron‐Cohen et al., 2001); the Social Aptitude Scale (SAS; Liddle et al., 2009); and the Social Responsiveness Scale (SRS‐2; Constantino & Gruber, 2012). Few studies (Tchanturia et al., 2016, 2021; Westwood et al., 2017, 2018) used diagnostic instruments such as the Autism Diagnostic Observation Schedule (ADOS‐2; Lord et al., 2012), the Autism Diagnostic Interview‐Revised (ADI‐R; Lord et al., 1994), or the Developmental Diagnostic Dimensional Interview‐short version (3Di‐sv; Santosh et al., 2009). One study used register diagnoses of autism from the Danish national health registers (Clausen et al., 2018).

## 2 AIMS

In this study we explore whether co‐occurring autism in patients with EDs is associated with greater ED symptom severity, ED service utilisation, and self‐harming behaviours. We expand on the existing literature by using a large cohort of individuals with ED (primarily clinically diagnosed AN), a broad range of ED severity indicators encompassing both registered clinical diagnoses and assessments, and self‐report data. To overcome the problem of assessing autistic traits during the acute phase of AN, we use valid measures of autism obtained through registered clinical diagnoses at any point in life. In addition, we avail ourselves of a novel approach to address the research questions on the genomic level by using autism polygenic scores (PGS) as an alternative measure of autism. We hypothesise that autism diagnosis and autism PGS will be associated with increased ED severity, in line with clinical observations and those studies that have observed such effects.

## 3 METHOD

### 3.1 Sample

**FIGURE 1:**
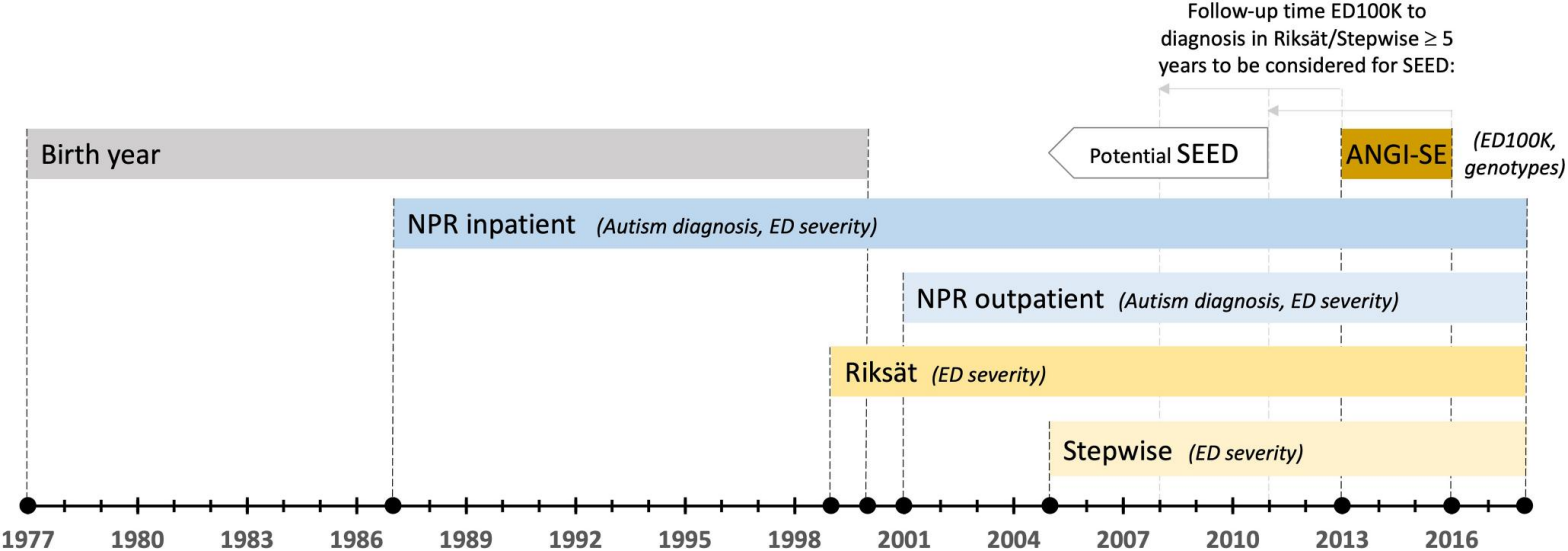
Overview of data sources used in the current study. ANGI‐SE: Anorexia Nervosa Genetics Initiative; ED: eating disorder; ED100K: questionnaire used in ANGI‐SE to determine lifetime anorexia nervosa diagnosis; NPR: national patient register; SEED: severe and enduring eating disorder

The Anorexia Nervosa Genetics Initiative (ANGI) is an international collaboration with a large sample of genotyped AN cases and controls (Thornton et al., 2018). From 2013 to 2016, case participants 16 years and older (i.e., born before 2000) from Sweden were recruited into ANGI (ANGI‐SE) via Swedish treatment centres and the national quality registers for specialised ED treatment *Riksät* (established in 1999) and *Stepwise* (established in 2005) (Birgegård et al., 2010). To be classified as a case in ANGI‐SE, participants had to have a DSM‐IV‐based lifetime AN diagnosis determined using answers to the ED100K‐v1 questionnaire (amenorrhoea was not required) (Thornton et al., 2018) or a clinical AN diagnosis registered in either Riksät/Stepwise (DSM‐IV‐TR; codes: 307.1, 307.5 criteria 1 and 2) or the National Patient Register (NPR; codes: ICD‐9: 307.1, ICD‐10: F50.0, F50.1). The NPR includes physician‐assigned diagnoses from inpatient care (ICD‐9 since 1987, ICD‐10 since 1997) and outpatient care (ICD‐10 since 2001) (Figure 1).

Our study population includes all ANGI‐SE cases born 1977–2000 with genotype data and available NPR linkage (to ascertain autism diagnoses). We excluded individuals born before 1977, as NPR data are only available from 1987 onwards; that is, from age 10 for the oldest ANGI‐SE participants born in 1977. Individuals who did not pass genotype quality control (i.e., with mismatched genetic and reported sex, with very low or high genetic heterogeneity, or with high familial relatedness) were also excluded. The final sample comprised 3189 AN cases (aged 16–39 at time of ED100K; 64 males) with register follow‐up through 31 December 2018.

### 3.2 Ethics

All participants provided written informed consent for participation in ANGI‐SE. ANGI‐SE was approved by the regional Ethical Review Board in Stockholm (Dnr 2013/112‐31/2, 2014/1563 and 2016/1852‐32).

### 3.3 Exposures

#### 3.3.1 Autism diagnosis

From the NPR, we extracted autism diagnoses using ICD codes (ICD‐9: 299A; ICD‐10: F84.0, F84.1, F84.5). In the absence of validation information for the autism diagnosis in the Swedish Registers, we required two occurrences of an autism diagnosis to increase confidence in the validity of the diagnoses and to screen out individuals who may have received a provisional diagnosis on one occasion that was not later confirmed. Individuals with only one registered autism diagnosis were included as controls.

#### 3.3.2 Autism PGS calculation

PGS for autism were computed for each participant using PLINK 2.0 and the summary statistics from the latest Psychiatric Genomics Consortium (PGC) genome‐wide association study (GWAS) of 18,381 individuals with autism and 27,969 controls as the discovery dataset (Grove et al., 2019). SNP quality control, including filtering for minor allele frequency (MAF > 0.10), removing duplicate SNPs, and matching with the discovery dataset, resulted in a subset of 221,241 SNPs. These were then clumped into clusters of approximate linkage disequilibrium (LD; *R* ^2^ < 0.1, within 500 kb distance) (Grove et al., 2019). The thresholding and clumping approach was used to generate PGS at 10 different *p*‐value thresholds (*p*‐values: 5 × 10^−8^, 1 × 10^−6^, 1 × 10^−4^, 0.001, 0.01, 0.05, 0.1, 0.2, 0.5, 1) (Choi et al., 2020). The final autism PGS used in this study was based on the first principal component (PGS‐PC1) from the principal component analysis (PCA) on the set of ten PGS. We included standardised PGS‐PC1 as a continuous variable, reporting results as one unit change per one standard deviation higher PGS (Coombes et al., 2020).

### 3.4 Outcomes

We extracted 29 outcomes in three groups (*ED severity & persistence, ED treatment received, Suicidal behaviour & self‐harm*) by combining available information from the ED100K (cross‐sectional and retrospective self‐reports), NPR (registered clinical diagnoses), and Riksät/Stepwise (registered clinical diagnoses, clinician ratings, and self‐reports at treatment start, during treatment, and at discharge) (Table 1). Whereas NPR only contains diagnostic codes along with the corresponding date and clinical setting (inpatient care vs. specialised outpatient care), Riksät/Stepwise provides greater detail on diagnostic subtypes (e.g., AN binge‐eating/purging type [AN‐BP]), as well as a wealth of clinical assessments and self‐reports. Riksät/Stepwise and NPR show moderate to excellent agreement for ED diagnoses. For example, 75% of individuals identified by the NPR as having AN also have an diagnosis in Riksät/Stepwise, and 91% of individuals identified by the NPR as *not* having AN also do *not* have AN in Riksät/Stepwise; the corresponding numbers for bulimia nervosa diagnosis are 84% and 91% (Birgegård et al., 2022).

**TABLE 1:** Overview of outcome definitions

Outcome	Source	Type	Comment
Outcome set 1: ED severity & persistence (11 outcomes)			
Age at ED onset	R/S (self), ED100K	Continuous	Earliest self‐reported age at first ED symptom. Onset before age 6 was treated as missing (*n*_missing_ = 334).
Age at first ED diagnosis	R/S (clin), NPR	Continuous	Earliest date of any registered ED diagnosis. ED diagnoses before age 6 were not considered (*n*_missing_ = 369).
Minimum adult BMI	ED100K	Continuous	Lowest weight (kg) since age 18 excluding times when physically ill/adult height^2 (m^2^)
Minimum BMI during AN	ED100K	Continuous	Lowest weight (kg) during periods of AN/corresponding height^2 (m^2^) at that time point. Age at lowest weight was not available; therefore, BMIs for individuals <18 years could not be corrected for age.
Maximum EDE‐Q global score	R/S (self)	Continuous (range: 0–6)	Scores were available at several time points for most individuals (e.g., during treatment period 1: At treatment initiation and at 1‐year follow‐up; during treatment period 2: At treatment initiation and at discharge). The lowest/highest (i.e., most severe) of all available values was selected. For individuals <18 years, CGAS instead of GAF was used.
Minimum GAF score	R/S (clin)	Continuous (range: 1–100)	
Maximum CIA score	R/S (self)	Continuous (range: 0–48)	
Maximum CGI score	R/S (clin)	Continuous (range: 1–7)	
Ever AN‐BP	R/S (clin)	Binary (yes/no)	Clinician‐assigned AN subtype. If ‘no’ AN subtype was always restrictive.
Ever OED	R/S (clin), NPR, ED100K	Binary (yes/no)	R/S: Clinician‐assigned diagnosis of bulimia nervosa or EDNOS
			NPR: Any primary or additional diagnosis of bulimia nervosa or EDNOS (ICD‐9: 307F, ICD‐10 F50.2, F50.3, F50.9)
			ED100K: Self‐reported bulimia nervosa
Severe and enduring ED	R/S (clin), ED100K	Binary (yes/no)	ANGI cases with any diagnosed ED in R/*S* ≥ 5 years ago and impairment by ED at time of ANGI survey (ED100K CIA score ≥ 18) were identified as having severe and enduring ED. Those with any diagnosed ED in R/*S* ≥ 5 years ago but with ED100K CIA score < 18 were identified as not having severe and enduring ED. This definition was previously used in Johansson et al., 2022.
Outcome set 2: ED treatment received (10 outcomes)			
Ever received ED treatment	R/S (clin), NPR	Binary (yes/no)	R/S: clinician‐assigned ED diagnosis within specialised ED; NPR: Inpatient records with a primary diagnosis of AN or OED
Ever outpatient with AN	NPR	Binary (yes/no)	NPR outpatient records with AN as primary or additional diagnosis
No. of outpatient visits with AN	NPR	Count	
Ever outpatient with OED	NPR	Binary (yes/no)	NPR outpatient records with OED as primary or additional diagnosis
No. of outpatient visits with OED	NPR	Count	
Ever inpatient with AN	NPR	Binary (yes/no)	NPR inpatient records with a primary diagnosis of AN
No. of inpatient days with AN	NPR	Count	
Ever inpatient with OED	NPR	Binary (yes/no)	NPR inpatient records with a primary diagnosis of OED
No. of inpatient days with OED	NPR	Count	
Ever received tube feeding	NPR	Binary (yes/no)	ICD‐10 procedure codes DJ010, DV065, TJD00, TJD10, TJD20, TJF10
Outcome set 3: Suicidal behaviour & self‐harm (8 outcomes)			
Any self‐harm	R/S (self)	Binary (yes/no)	Includes self‐harm behaviours without suicidal intent. The period with the highest frequency of self‐harm behaviours was selected (i.e., the most severe period).
Frequency of self‐harm when most severe	R/S (self)	Ordinal (never, 1‐2x, 3‐10x, 11‐100x, >100x)	
Any suicidal thoughts/plans	R/S (self)	Binary (yes/no)	
Frequency of suicidal thoughts/plans	R/S (self)	Ordinal (never, a few times in life, ≥1/week for a 3‐month period)	
Any documented suicide attempt	NPR	Binary (yes/no)	NPR records of suicide attempts (ICD‐9 E950‐E959, E980‐E989; ICD‐10 X60‐X84, Y10‐Y34)
No. of documented suicide attempts	NPR	Count	
Any self‐reported suicide attempt with ED	R/S (self)	Binary (yes/no)	Self‐reported suicide attempts within the last 12 months of treatment initiation or within the last 12 months since last R/S follow‐up. Since all reported suicide attempts are reported at initiation of/during/fat discharge from of ED treatment, they are considered as ‘with ED’.
Frequency of self‐reported suicide attempts	R/S (self)	Ordinal (never, 1‐2x, ≥3x)	Due to the way the variable was coded in R/S, we were not able to calculate the exact number of self‐reported suicide attempts. This outcome therefore represents a lower bound of self‐reported suicide attempts.

Abbreviations: AN‐BP, AN binge‐purging subtype; ANGI, Anorexia Nervosa Genetics Initiative; BMI, body mass index; CGAS, Children's Global Assessment Scale; CGI, Clinical Global Impression; CIA, Clinical Impairment Assessment; ED, eating disorder; EDE‐Q, Eating Disorder Examination‐Questionnaire; EDNOS, eating disorder not otherwise specified; ED100K, survey used in ANGI; GAF, Global Assessment of Functioning; NPR, national patient register; OED, other eating disorder; R/S, Riksät/Stepwise (quality register); R/S (clin), based on clinical diagnosis or clinician rating; R/S (self), based on self‐report.

In Riksät/Stepwise, the same variables are assessed at multiple time points during each treatment episode an individual has (treatment initiation, yearly follow‐up during ongoing treatment, discharge). For example, one individual completed the EDE‐Q at treatment initiation, 1‐year follow‐up, and discharge of treatment episode 1, as well as at treatment initiation of treatment episode 2. Although both initial and follow‐up assessments are mandated in most of the ED clinics reporting to Riksät/Stepwise, it is common that yearly follow‐up and discharge assessments are not conducted (i.e., not registered), whereas missingness is low for assessments at treatment initiation (Birgegård et al., 2010). To capture ED severity, we chose the highest/lowest (i.e., most severe) score ever reported for an individual where applicable: maximum EDE‐Q global score, maximum Clinical Impairment Assessment (CIA) score (Bohn et al., 2008), minimum Global Assessment of Functioning (GAF) score (American Psychiatric Association, 2000), maximum Clinical Global Impression (CGI) score (Guy, 2000). Table 1 provides a detailed overview of the source and definition of each outcome.

### 3.5 Statistical analysis

Analyses were performed using R version 4.0.5. We used logistic regression (*drgee* package) for binary outcomes, linear regression (*drgee* package) for continuous outcomes, Poisson regression (*gee* package) for count outcomes (with robust sandwich estimator to account for zero‐inflation), and ordinal logistic regression (*MASS* package) for ordinal outcomes. Sex and birth year in 3‐year intervals (i.e., 1977–1979, etc.) were included as covariates in all models. For autism PGS analysis, we also adjusted for the first 10 genetic ancestry principal components. We report odds ratios (OR), standardised and unstandardised beta estimates (*β* and *B*), and incidence rate ratios (IRR) dependent on outcome types, including the 95% confidence intervals (CI). We applied false discovery rate correction for dependent outcomes according to Benjamini and Yekutieli (2001) to account for multiple testing (*q* < 0.05). Although four models per outcome were computed (autism diagnosis not adjusted/adjusted for other neurodevelopmental disorders [NDDs], autism PGS not adjusted/adjusted for other NDDs), each of the four models per outcome tested the same hypothesis that autism is associated with ED severity; we therefore only corrected for one model per outcome (i.e., 29 tests altogether).

### 3.6 Sensitivity analyses

Autism often co‐occurs with other NDDs such as attention deficit hyperactivity disorder (ADHD) and intellectual disability, which themselves are associated with ED and many other negative outcomes (Du Rietz et al., 2017; Yao et al., 2019). To determine whether any effects on ED severity outcomes are due to autism rather than to other comorbid NDDs, we included NDD as an additional covariate for sensitivity analysis. From the NPR, we extracted ICD codes for ADHD (ICD‐9: 314; ICD‐10: F90) and intellectual disability (ICD‐9: 317–319, ICD‐10: F70‐F79). The presence of ADHD and/or intellectual disability indicated having an NDD. Furthermore, since individuals with only one registered autism diagnosis were treated as controls in the main analyses, we also conducted sensitivity analyses excluding these individuals from the analyses (i.e., treated as neither cases nor controls).

## 4 RESULTS

The total sample consisted of 3189 individuals (64 males) with current or previous AN. Among these, we identified 134 (4.2%) individuals who were diagnosed with autism at least twice. The proportion of individuals diagnosed with autism did not differ significantly across birth year categories (range: 3.0%–6.1%; *χ* ^2^(7) = 10.64, *p* = 0.155). The median age at first autism diagnosis was 23 years (range 11–38 years), but differed significantly depending on birth year category (one‐way ANOVA: *F*(7) = 31.66, *p* < 2 × 10^−16^) with those born earliest (1977–1979) having the highest median age (34 years, range: 27–38 years) and those born latest (1998–2000) having the lowest median age (17 years, range 14–19 years; Table S1). A substantial majority (86%) received their first autism diagnosis (NPR) *after* their first ED diagnosis (NPR or Riksät/Stepwise)—on average 6.1 years later (median: 5 years, *interquartile range*: 6 years). Autism PGS explained 1.82% (Nagelkerke's *R* ^2^) of the variance of autism diagnosis and was not statistically significantly higher in individuals diagnosed with autism (Table 2).

**TABLE 2:** Study population characteristics

		Without autism,	With autism,
Characteristic	*N*	*N* = 3055	*N* = 134
Autism PGS, mean (SD)	3189	−0.01 (0.99)	0.05 (1.03)
Birth year, mean (SD)	3189	1988.77 (5.54)	1989.04 (5.28)
Sex, *n*/*N* (%)	3189		
Male		57/3055 (1.9%)	7/134 (5.2%)
Female		2998/3055 (98%)	127/134 (95%)
Outcome set 1: ED severity & persistence			
Age at ED onset, mean (SD)	2855	14.51 (3.11)	13.65 (3.77)
(Missing)		333	1
Age at first ED diagnosis, mean (SD)	2820	18.87 (4.36)	18.45 (4.52)
(Missing)		366	3
Minimum adult BMI, mean (SD)	2678	16.70 (2.39)	15.58 (2.69)
(Missing)		486	25
Minimum BMI during AN ever, mean (SD)	2945	15.59 (1.87)	14.76 (2.34)
(Missing)		226	18
Maximum EDE‐Q global score, mean (SD)	1816	3.70 (1.36)	4.30 (1.31)
(Missing)		1332	41
Minimum GAF score, mean (SD)	2729	45.93 (13.12)	35.84 (13.27)
(Missing)		452	8
Maximum CIA score, mean (SD)	1307	29.61 (11.59)	33.62 (12.34)
(Missing)		1822	60
Maximum CGI score, mean (SD)	1085	4.31 (1.52)	5.28 (1.09)
(Missing)		2024	80
Ever AN‐BP, *n*/*N* (%)	2758	350/2632 (13%)	25/126 (20%)
(Missing)		423	8
Ever OED, *n*/*N* (%)	3189	2336/3055 (76%)	125/134 (93%)
Severe and enduring ED, *n*/*N* (%)	1147	402/1098 (37%)	33/49 (67%)
(Missing)		1957	85
Outcome set 2: ED treatment received			
Ever received ED treatment, *n*/*N* (%)	3189	2632/3055 (86%)	126/134 (94%)
Ever outpatient with AN, *n*/*N* (%)	3189	1551/3055 (51%)	95/134 (71%)
No. of outpatient visits with AN, mean (SD)	3189	6.04 (14.59)	17.34 (27.13)
Ever outpatient with OED, *n*/*N* (%)	3189	1645/3055 (54%)	108/134 (81%)
No. of outpatient visits with OED, mean (SD)	3189	4.37 (14.23)	8.67 (10.84)
Ever inpatient with AN, *n*/*N* (%)	3189	698/3055 (23%)	62/134 (46%)
No. of inpatient days with AN, mean (SD)	3189	28.36 (110.12)	121.90 (393.36)
Ever inpatient with OED, *n*/*N* (%)	3189	259/3055 (8.5%)	35/134 (26%)
No. of inpatient days with OED, mean (SD)	3189	4.41 (23.77)	17.89 (53.13)
Ever received tube feeding, *n*/*N* (%)	3189	61/3055 (2.0%)	13/134 (9.7%)
Outcome set 3: Suicidal behaviour & self‐harm			
Any self‐harm, *n*/*N* (%)	1803	723/1708 (42%)	65/95 (68%)
(Missing)		1347	39
Frequency of self‐harm when most severe, *n*/*N* (%)	1803		
Never		985/1708 (58%)	30/95 (32%)
1–2 times		105/1708 (6.1%)	5/95 (5.3%)
3–10 times		225/1708 (13%)	12/95 (13%)
11–100 times		306/1708 (18%)	33/95 (35%)
More than 100 times		87/1708 (5.1%)	15/95 (16%)
(Missing)		1347	39
Any suicidal thoughts/plans, *n*/*N* (%)	1106	420/1051 (40%)	38/55 (69%)
(Missing)		2004	79
Frequency of suicidal thoughts/plans, *n*/*N* (%)	1106		
Never		631/1051 (60%)	17/55 (31%)
A couple of times in their life		239/1051 (23%)	17/55 (31%)
At least one period with suicidal thoughts/plans at least once per week		181/1051 (17%)	21/55 (38%)
(Missing)		2004	79
Any documented suicide attempt, *n*/*N* (%)	3189	373/3055 (12%)	56/134 (42%)
No. of documented suicide attempts, mean (SD)	3189	0.60 (4.71)	4.85 (12.82)
Any self‐reported suicide attempt with ED, *n*/*N* (%)	787	59/743 (7.9%)	11/44 (25%)
(Missing)		2312	90
Frequency of self‐reported suicide attempts, *n*/*N* (%)	1102		
Never		870/1047 (83%)	36/55 (65%)
1–2 times		119/1047 (11%)	9/55 (16%)
3 times or more		58/1047 (5.5%)	10/55 (18%)
(Missing)		2008	79

### 4.1 Autism diagnosis and ED severity

**FIGURE 2:**
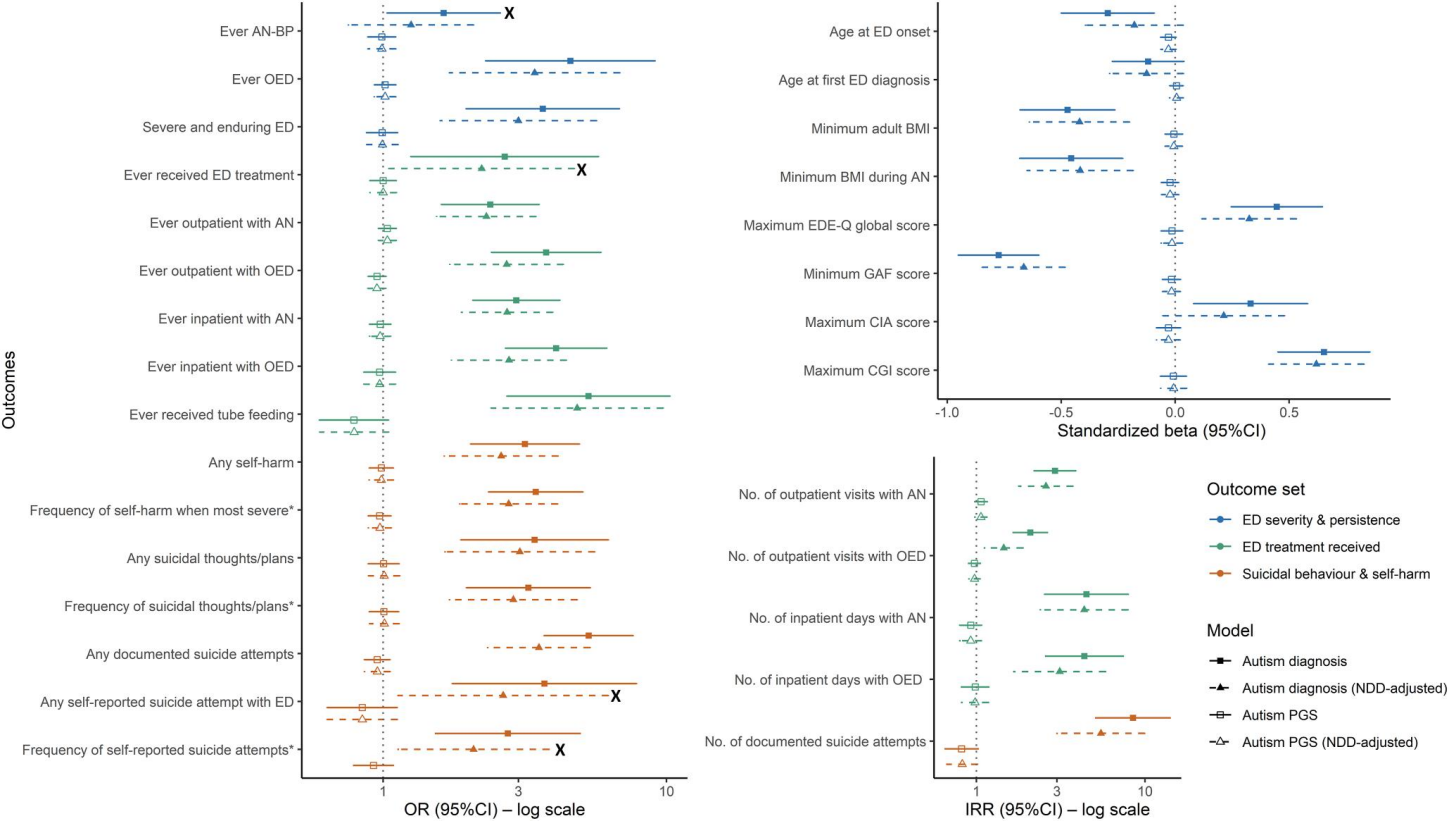
Effect size with 95% CI for the association of autism diagnosis/autism PGS with 29 ED severity indicators in ANGI‐SE AN cases. Effect sizes from regression models are plotted separately by type of effect size (odds ratios [OR] for logistic and ordinal (labelled with ∗ next to the outcome names) regressions, standardised beta for linear regressions, and incidence rate ratios [IRR] for Poisson regressions). Different colours represent different outcome groups (blue: ED severity & persistence; green: ED treatment received; orange: Suicidal behaviour & self‐harm). Different shapes of the point estimates and different line types represent the different models. Black crosses (X) label the tests which were initially significant but did not pass false discovery rate correction. Note: NDD‐adjusted autism PGS analysis was not applicable for the outcome *Frequency of self‐reported suicide attempts* due to insufficient power

Out of 29 tests, 27 tests were statistically significant between the autism and no autism groups. All significant effects showed the expected direction (higher severity in individuals with autism). Table 2 includes descriptive statistics for all outcomes by autism group. Figure 2 depicts the corresponding effects sizes and 95% CIs. Exact effect size values and unstandardised beta estimates for linear regressions can be found in Table S2.

#### 4.1.1 Outcome set 1: ED severity & persistence

Autism diagnosis was statistically significantly associated with increased severity for all outcomes in this outcome set, except *age at first ED diagnosis* and ever being diagnosed with *AN‐BP* (vs. AN‐restricting type only). For binary outcomes, ORs ranged from 1.64 (95% CI 1.03–2.59) for *ever AN‐BP* to 4.58 (95% CI 2.30–9.10) for *ever OED*. For continuous outcomes, the lowest absolute *β* estimate was seen for *age at first ED diagnosis* (−0.12, 95% CI −0.28 to 0.04); the highest absolute *β* estimate was seen for *minimum GAF score* (−0.77, 95% CI −0.95 to −0.60). Compared to individuals without autism, those with diagnosed autism had more severe ED symptoms, including a 0.6‐point higher *maximum EDE‐Q global score* (theoretical range: 0–6) and ∼1 kg/m^2^ lower *minimum adult BMI* and *minimum BMI during AN*. The autism group was also characterised by higher clinical impairment and lower functioning, as indexed by a 4‐point higher *maximum CIA score* (theoretical range: 0–48), a ∼1‐point higher *maximum CGI score* (theoretical range: 1–7), and a ∼10‐point lower *minimum GAF score* (theoretical range: 1–100). Although *age at first ED diagnosis* did not differ significantly between people with and without autism, the *age at ED onset* was on average 1 year earlier for individuals with autism. Almost all individuals with autism (93%) were diagnosed with *OED* at some point in their life, compared to 76% of individuals without autism (OR 4.58 [95% CI 2.30–9.10]). Strikingly, almost two thirds (67%) of those with autism developed *severe and enduring ED* (defined by ED duration of ≥5 years & ED100K CIA score ≥18), compared to one third (37%) of those without autism. This corresponded to ∼4 times higher odds of severe and enduring ED in the autism group.

#### 4.1.2 Outcome set 2: ED treatment received

Autism diagnosis was statistically significantly associated with all 10 outcomes in this group. Overall, duration and intensity of treatment received was considerably higher in the autism group. Among the binary outcomes, ORs ranged from 2.39 (95% CI 1.61–3.55) for *ever outpatient with AN* to 5.31 (95% CI 2.74–10.29) for *ever received tube feeding*. Among the count outcomes, IRRs ranged from 2.09 (95% CI 1.65–2.63) for *number of outpatient days with OED* to 4.49 (95% CI 2.54–7.94) for *number of inpatient days with AN*. Almost half (46%) of the autism group received inpatient treatment for AN (vs. 23% of those without autism), corresponding to three times higher odds of receiving inpatient care for AN in individuals with autism. The *number of inpatient days with AN* was markedly greater in the autism group who spent 122 days in inpatient care on average (vs. 28 days in the group without autism; IRR = 4.49, 95% CI 2.54–7.94). A similar pattern emerged for the *number of inpatient days with OED* (18 vs. 4 days; IRR = 4.37, 95% CI 2.57–7.41).

#### 4.1.3 Outcome set 3: Suicidal behaviour & self‐harm

All 8 outcomes differed significantly between groups. The autism group had significantly higher odds of self‐harm behaviours, suicidal thoughts/plans, and suicide attempts, as well as higher frequencies of these behaviours. For binary and ordinal outcomes, ORs ranged from 2.75 (95% CI 1.53–4.95) for *frequency of self‐reported suicide attempts* to 5.31 (95% CI 3.70–7.63) for *any documented suicide attempts.* IRR was 8.47 (95% CI 5.10–14.05) for *number of documented suicide attempts*—the only count outcome in this set. Among individuals with autism, 16% reported having harmed themselves more than 100 times during the most severe period of self‐harm in their life (vs. 5% in individuals without autism; OR = 3.46, 95% CI 2.35–5.07). Close to half (42%) of individuals with autism had at least one *documented suicide attempt* (vs. 12% of individuals without autism). The *number of documented suicide attempts* was ∼8 times higher in individuals with autism (on average the autism group committed ∼5 suicide attempts). The autism group also had ∼4 times higher odds to report at least one *suicide attempt with ED* (i.e., within the last 12 months of ED treatment start or within the last 12 months since last follow‐up in Riksät/Stepwise).

### 4.2 Autism PGS and ED severity

Autism PGS was not statistically significantly associated with any of the outcomes, and most effect sizes were close to zero/one (OR range: 0.79–1.02, *β* range: −0.34–0.03, IRR range: 0.82–1.06).

### 4.3 Sensitivity analyses

Controlling for coexisting NDDs (including ADHD and intellectual disability) generally decreased the effects of autism diagnosis on ED severity; however, most of these reductions in effect size were small (range of reduction: 0%–39% of the original effects size) and the confidence intervals of NDD‐unadjusted and ‐adjusted models were highly overlapping. Twenty‐two of 29 outcomes were significantly associated with ED severity when adjusting for NDDs (Figure 2), that is, five outcomes fewer than in the NDD‐unadjusted analysis *(ever received ED treatment, any self‐reported suicide attempt with ED, age at ED onset*, *maximum CIA score,* and *frequency of self‐reported suicide attempts)*. As for the NDD‐unadjusted results, none of the outcomes was significantly associated with autism PGS when adjusting for NDDs. Excluding individuals with only one autism diagnosis in the NPR (*n* = 11) from the non‐autism group in the sensitivity analyses yielded almost identical results as the main analyses (Table S3 & Figure S1).

## 5 DISCUSSION

This study examined the hypothesis that individuals with co‐occurring autism have greater ED severity than those without co‐occurring autism, using a broad range of ED severity indicators in a large cohort of individuals with lifetime AN. Co‐occurring autism was operationalised using registered autism diagnoses, and analyses were repeated using autism PGS. Overall, our results provide strong evidence for our hypothesis: confirmed autism diagnosis was associated with higher ED severity on almost all investigated severity indicators. In contrast, the autism PGS was *not* statistically significantly associated with any of the outcomes. Below, we first discuss the representativeness of our sample for the population with ED with or without autism. Second, we review our results for autism diagnosis and autism PGS with reference to previous studies and discuss possible reasons for discrepant findings.

The prevalence of autism in our sample of individuals with lifetime AN (4.2%) is at the lower end of previous estimates (Westwood & Tchanturia, 2017), which is unsurprising considering that we used clinical diagnoses and required at least two records. Previous studies might have overestimated autism prevalence in AN due to confounded cross‐sectional measurements of autistic traits during the acute stage of illness (e.g., confounded by the effects of starvation). Autism prevalence in our sample is comparable to the Swedish population with EDs. In a study of all individuals born 1977–2003 in Sweden with an ED registered either in the NPR or in Riksät/Stepwise (*n*_AN_ = 12,424, *n*_OED_ = 20,716; 94% female), Zhang et al. (2021) found registered autism diagnoses in 3.5% of individuals with AN and in 4.1% of individuals with OED. By exclusively relying on NPR‐registered autism diagnosis we can be certain that our sample includes at least some false negatives, especially since our sample was mainly female and autism is often missed in females (Lai & Szatmari, 2020). The inclusion of false negatives in the group without autism would likely have influenced our results towards the null. Furthermore, whereas autism diagnoses are most often given in outpatient care, the outpatient register was only available from 2001 onwards (i.e., when the oldest individuals in our sample were 24 years old) and first reached full coverage in 2010. However, although the age at first diagnosis with autism differed significantly by birth year (higher in older individuals), there was no trend for lower prevalence of diagnosed autism among older individuals (Table S1).

The age of first autism diagnosis was very high in this sample (minimum: 11 years, median: 23 years), which might at least partly be explained by the fact that the sample comprised primarily females. It is well‐known that mechanisms such as the nature of symptomatology of female autism (Hiller et al., 2014) and camouflaging (i.e., coping strategies to hide autistic behaviours or perform behaviours considered as neurotypical to socially fit in) (Lai et al., 2017) contribute to late or missed autism diagnosis in females. In addition, psychiatric conditions such as EDs, anxiety, and depression can obscure an underlying autism and delay its diagnosis so that autism is often only suspected when treating these disorders remains unsuccessful (Mandy & Tchanturia, 2015; Trubanova et al., 2014). In line with this, 86% of individuals in our sample received their first autism diagnosis *after* their first ED diagnosis. Furthermore, the age of first autism diagnosis in our sample was similarly high as that in a population‐based sample of ∼6000 Swedish twins aged 17–24 years, where the 31 females with autism were first diagnosed at a median age of 17.3 years (range: 3.5–22.1 years), with only two of them being diagnosed before age 10 years (Dinkler et al., 2021). Our finding is also in line with a study using the Danish National Patient Registry, reporting that EDs were much more commonly diagnosed in individuals diagnosed with autism in late childhood (11–15 years) compared to early or mid‐childhood (Rødgaard et al., 2021).

Nevertheless, the late age of diagnosis raises the question whether our sample only includes milder or otherwise distinct cases of autism. Participation in ANGI‐SE required individuals to complete questionnaires and contribute a blood sample, and it is therefore likely that individuals with more severe autism (e.g., with co‐occurring intellectual disability), which is usually detected early, did not participate in ANGI‐SE. The prevalence of AN, bulimia nervosa, and binge‐eating disorder in individuals with severe autism/intellectual disability is not well‐known, mainly limited to case reports, and often related to genetic syndromes (Gritti et al., 2011) indicating that their overlap is not very common. Thus, although our sample might not fully reflect the heterogeneity of the population with autism, we believe that it is relatively representative of individuals with autism and AN, bulimia nervosa, binge‐eating disorder, or ED not otherwise specified.

### 5.1 Autism diagnosis and ED severity

Contrary to published studies, we found greater severity of core ED symptoms (frequency of ED thoughts and behaviours measured with EDE‐Q, and BMI) in individuals with autism. Previous null findings might have been due to low power due to small sample sizes in addition to predominantly cross‐sectional measurement of autistic traits, leading to an overclassification of individuals with EDs and autism/high autistic traits, whereas the actual prevalence of individuals with autism in these samples may have provided too low power to find significant differences in core ED symptoms. In line with previous studies (Nazar et al., 2018; Stewart et al., 2017; Tchanturia et al., 2021), we found that individuals with co‐occurring autism received inpatient treatment for ED more frequently and had longer durations of treatment. Our results also align with the previously reported observation of greater likelihood of involuntary treatment such as tube feeding in people with co‐occurring autism (Clausen et al., 2018).

Furthermore, it appeared that the average *age at ED onset* (measured as the self‐reported age at first ED symptom) was 1 year earlier for individuals with autism, whereas the *age at first ED diagnosis* was roughly equal across groups. Although self‐reported age at first ED symptom is likely influenced by recall bias, it is not clear why any bias should differ between the group with and the group without autism. Therefore, this result implies that people with ED and autism might have a greater duration of untreated ED, which could partly explain their worse clinical outcomes.

In line with previous studies reporting lower overall functioning among individuals with ED and autism in the long‐term (often long after ED recovery), we found that autism was also associated with lower overall functioning during episodes with active ED, as evidenced by GAF, CGI, and CIA scores. We argue, however, that lower overall functioning is not necessarily a good indicator of greater ED severity in individuals who also have autism. That is because autism itself is defined by impaired social functioning, and further, autism is associated with increased risk of other psychiatric disorders (Lai et al., 2019), which in turn might further impair functioning. Accordingly, the differences between individuals with and without autism in our sample were larger for more general measures of functioning/impairment (GAF, CGI), and somewhat lower for impairment specific to ED (CIA). In fact, after adjusting for other NDDs, the difference in CIA score became non‐significant, supporting the notion that lower functioning/higher impairment in the autism group might be less of an indicator of ED severity, but rather more reflective of autism and other NDD comorbidity (i.e., an ED‐unspecific outcome).

### 5.2 Autism PGS and ED severity

Contrary to our hypothesis, no significant associations with ED severity were found for autism PGS. The most likely explanation for this unexpected finding is that the current autism PGS was too low‐powered. In the latest autism GWAS (from which summary statistics were used to compute autism PGS for the current study), autism PGS explained 2.45% of the variance of case‐control status (Grove et al., 2019), which is comparable to the PGS for major depressive disorder (explained variance 1.5%–3.2%) (Howard et al., 2019), but less strong than the PGS for ADHD (explained variance 5.5%) (Demontis et al., 2019) and schizophrenia (explained variance 7.3%) (Trubetskoy et al., 2022). In our sample, autism PGS explained 1.8% of the variance in autism diagnostic status, which is somewhat lower than in the original GWAS, and furthermore, autism PGS was not significantly higher in individuals with autism diagnosis compared to those without (*p* = 0.46) in our study population, indicating low power. However, other studies based on the same discovery GWAS also found that autism PGS was only weakly related or even unrelated to autism diagnosis and psychiatric family history of autism (Jansen et al., 2020; Schendel et al., 2022). That rare genetic variants play an important role in the aetiology of autism (Geschwind & State, 2015) might be an explanation for the weakness of the current autism PGS which only captures effects from common variants. In our sample, the low power of the autism PGS might have been further eroded by the low proportion of autism cases (4.2%) in our sample compared to the 1:1.5 case‐control ratio in the discovery GWAS—a recent study has shown that the proportion of phenotypic variance explained by a PGS increases with increasing proportion of cases in the sample (Trubetskoy et al., 2022).

Other explanations for the non‐significant associations between autism PGS and ED severity might be the large differences in sex distribution and age at first autism diagnosis between our sample and the discovery GWAS used to derive autism PGS, potentially indicating that slightly different autism phenotypes were captured. Although the sex distributions in our sample (95% females) and the autism GWAS sample (∼20% females) are largely in line with the inversely skewed sex ratios of AN and autism (Loomes et al., 2017; Murray et al., 2017), there is some evidence that females need greater familial etiologic load than males (including higher mutational burden), in order to manifest the autism phenotype (Wigdor et al., 2022); the opposite has been suggested for AN in males, who need greater familial etiologic load in to be diagnosed (Steinhausen et al., 2015)). In the autism literature, this has been discussed as the *female protective effect*, though this effect could also be due to diagnostic bias (i.e., worse detection of female autism), which is supported by the finding that females need to express more symptoms to be diagnosed (Lundström et al., 2019). A similar mechanism might apply for males with AN, who are often underrecognized (Murray et al., 2017). Lastly, and as discussed above, the high age at first autism diagnosis in our sample suggests that individuals with more severe autism (i.e., those who are likely to have a high autism PGS), did not participate in ANGI‐SE. In summary, our results regarding the association of autism PGS with ED severity are inconclusive; however, with larger GWAS producing more refined autism PGS, we do expect to find such associations in the future.

### 5.3 Strengths and limitations

The major strength of the current study is the large sample of individuals with lifetime AN and with deep phenotyping for a broad range of ED outcomes gathered from different sources including national registers and survey data. Furthermore, our rigorous definition of autism (at least two registered diagnoses), overcomes a major limitation of previous studies by minimising the risk of confusing autistic traits with symptoms related to AN and starvation. Nonetheless, the high age of onset for autism in our sample suggests possible differences in cases reported here and those from which the autism PGS was derived. Unfortunately, we do not have additional data on autism symptoms or course to further explore this possibility, nor to determine whether individuals with more severe AN might simply be more likely to be given a diagnosis of comorbid autism during acute phases of illness. It is also possible that our sole reliance on NPR‐registered autism diagnoses might have led to an under‐identification of individuals with autism (false negatives), which would likely have reduced differences between groups (i.e., biased our results towards the null). Finally, the proportion of males in our sample was very low (5%); therefore, our conclusions apply almost exclusively to women with AN. Future studies need to increase efforts to recruit males with EDs for research.

## 6 CONCLUSION

Our findings provide strong confirmation of the clinical observation that individuals with *diagnosed* autism experience higher ED severity. Some of the largest differences were found for time spent in inpatient care and proportion of individuals who received tube feeding. In addition, individuals with co‐occurring autism are at highly increased risk of (multiple) suicide attempts. Results regarding autism PGS were inconclusive. These findings suggest that individuals with co‐occurring autism need extra attention, and efforts to adapt ED treatment to improve treatment outcome (Li et al., 2021; Tchanturia et al., 2020) are therefore of high importance. ED clinicians should also be aware of the higher risk of self‐harm and suicidal behaviours among individuals with autism.

## AUTHOR CONTRIBUTIONS

Ruyue Zhang: conceptualisation, methodology, software, validation, formal analysis, data curation, writing – original draft preparation, review & editing, visualisation. Andreas Birgegård: resources, writing – review & editing. Bengt Fundín: writing – review & editing. Mikael Landén: investigation, resources, writing – review & editing. Laura M. Thornton: investigation, writing – review & editing. Cynthia M. Bulik: conceptualisation, investigation, resources, writing – review & editing, funding acquisition; Lisa Dinkler: conceptualisation, methodology, validation, data curation, writing – original draft preparation, review, & editing, visualisation, supervision.

## CONFLICTS OF INTEREST

Cynthia M. Bulik reports: Shire (grant recipient, Scientific Advisory Board member); Lundbeckfonden (grant recipient); Pearson (author, royalty recipient). Mikael Landén declares that he has received lecture honoraria from Lundbeck pharmaceutical. All other authors have indicated they have no conflicts of interest to disclose.

## Supporting information

Figure S1

Supplementary Material

Table S1

Table S1

Table S2

Table S3

## Data Availability

The data that support the findings of this study are available on request from the corresponding author. The data are not publicly available due to privacy or ethical restrictions.
