# Supplementary figures and images for: Association of autism diagnosis and polygenic scores with eating disorder severity
Source: Eur Eat Disord Rev. 2022 Jul 19;30(5):442–58. doi: 10.1002/erv.2941 (PMC9544642; doi:10.1002/erv.2941)

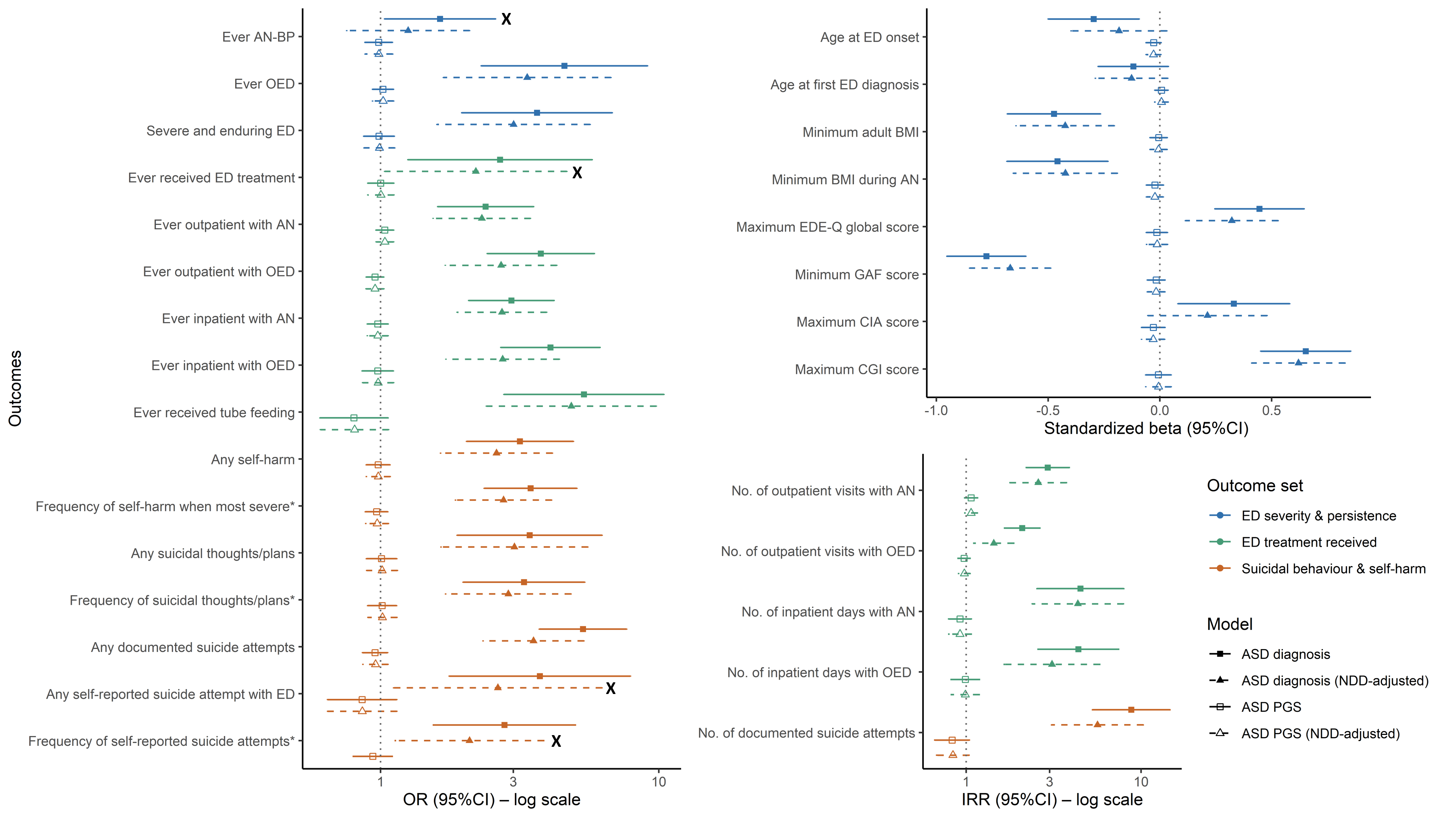

Supplement: Supplementary file 1 — Figure S1 [file ERV-30-442-s003.png]
