# Supplementary material for: Association of autism diagnosis and polygenic scores with eating disorder severity
Source: Eur Eat Disord Rev. 2022 Jul 19;30(5):442–58. doi: 10.1002/erv.2941 (PMC9544642; doi:10.1002/erv.2941)
Supplement: Supplementary file 2 — Supplementary Material [file ERV-30-442-s001.docx]

**Supplementary information** to Zhang et al. - Association of autism diagnosis and polygenic scores with eating disorder severity

**Table S1**: Prevalence of autism spectrum disorder (autism) and age at first autism diagnosis in the study population by birth year category

|  | | |
| --- | --- | --- |
| Birth year category, N | **No. with autism diagnosis** | **Age at first autism diagnosis, Median (range)** |
| [1977,1979], N=190 | 6 (3.2%) | 34 (27,38) |
| [1980,1982], N=314 | 11 (3.5%) | 30 (24,37) |
| [1983,1985], N=396 | 14 (3.5%) | 30 (23, 33) |
| [1986,1988] , N=576 | 35 (6.1%) | 26 (16, 32) |
| [1989,1991] , N=619 | 21 (3.4%) | 22 (15, 28) |
| [1992,1994] , N=533 | 26 (4.9%) | 18 (15, 26) |
| [1995,1997] , N=430 | 13 (3.0%) | 17 (11, 21) |
| [1998,2000] , N=131 | 8 (6.1%) | 17 (14, 19) |

**Table S2**: Point estimates and 95% confidence intervals displayed in Figure 2

*See separate excel file*

**Table S3**: Point estimates and 95% confidence intervals displayed in Figure S1

*See separate excel file*


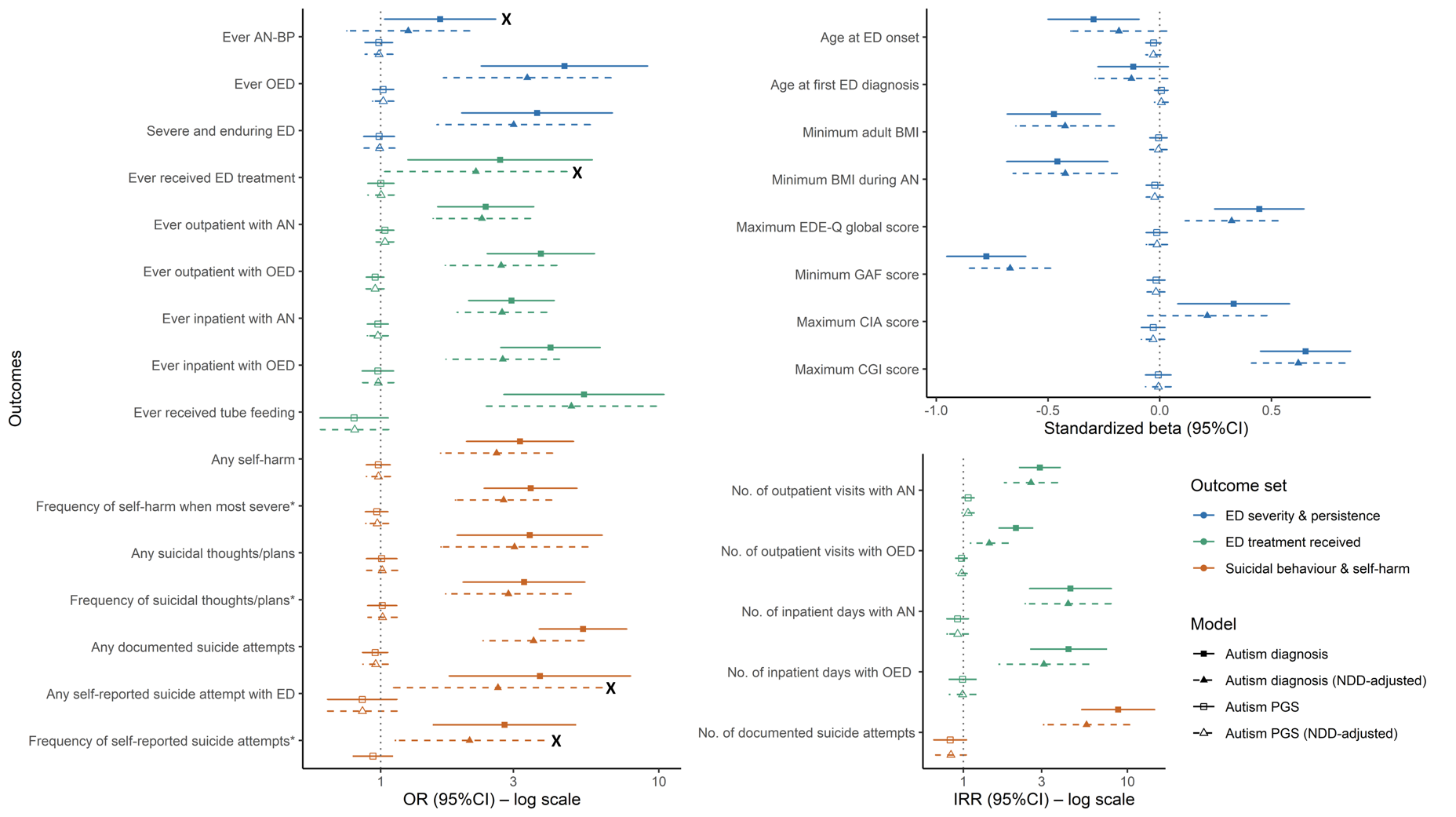


**Figure S1**: Effect size with 95% CI for the association of autism diagnosis/autism PGS with 29 ED severity indicators in ANGI-SE AN cases, excluding 11 individuals with only one autism diagnosis

Effect sizes from regression models are plotted separately by type of effect size (odds ratios [OR] for logistic and ordinal (labelled with * next to the outcome names) regressions, standardised beta for linear regressions, and incidence rate ratios [IRR] for Poisson regressions). Different colours represent different outcome groups (blue: ED severity & persistence; green: ED treatment received; orange: Suicidal behaviour & self-harm). Different shapes of the point estimates and different line types represent the different models. Black crosses (X) label the tests which were initially significant but did not pass false discovery rate correction. Note: NDD-adjusted autism PGS analysis was not applicable for the outcome *Frequency of self-reported suicide attempts* due to insufficient power.
