# Supplementary material for: Association of autism diagnosis and polygenic scores with eating disorder severity
Source: Eur Eat Disord Rev. 2022 Jul 19;30(5):442–58. doi: 10.1002/erv.2941 (PMC9544642; doi:10.1002/erv.2941)
Supplement: Supplementary file 3 — Table S1 [file ERV-30-442-s004.docx]

| Table S1. Prevalence of autism diagnosis and age at first autism diagnosis in the study population by birth year category | | |
| --- | --- | --- |
| Birth year category, N | No. with autism diagnosis | Age at first autism diagnosis, Median (range) |
| [1977,1979], N=190 | 6 (3.2%) | 34 (27,38) |
| [1980,1982], N=314 | 11 (3.5%) | 30 (24,37) |
| [1983,1985], N=396 | 14 (3.5%) | 30 (23, 33) |
| [1986,1988] , N=576 | 35 (6.1%) | 26 (16, 32) |
| [1989,1991] , N=619 | 21 (3.4%) | 22 (15, 28) |
| [1992,1994] , N=533 | 26 (4.9%) | 18 (15, 26) |
| [1995,1997] , N=430 | 13 (3.0%) | 17 (11, 21) |
| [1998,2000] , N=131 | 8 (6.1%) | 17 (14, 19) |
